# 6,7,4′-Trihydroxyflavanone Protects against Dextran Sulfate Sodium-Induced Colitis by Regulating the Activity of T Cells and Colon Cells In Vivo

**DOI:** 10.3390/ijms22042083

**Published:** 2021-02-19

**Authors:** Hyun-Su Lee, Gil-Saeng Jeong

**Affiliations:** College of Pharmacy, Keimyung University, Daegu 42601, Korea; hyunsu.lee@kmu.ac.kr

**Keywords:** 6,7,4′-trihydroxyflavanone, colitis, T cell activation, colon cells, NF-κB, MAPK

## Abstract

Colitis is a multifactorial disorder that mostly occurs in the gastrointestinal tract. Despite improvements in mucosal inflammation research, little is known regarding the small bioactive molecules that are beneficial for regulating T cells and colon cell activity. 6,7,4′-trihydroxyflavanone (THF) is a flavanone that possesses anti-osteoclastogenesis activity and exerts protective effects against methamphetamine-induced immunotoxicity. Whether THF mitigates intestinal inflammation by regulating T cells and colon cell activity remains unknown. In the present study, Jurkat and HT-29 cells were used for in vitro experiments, and dextran sulfate sodium (DSS)-induced colitis model in mice was used for in vivo experiment. We observed that THF did not have a negative effect on the viability of Jurkat and HT-29 cells. Quantitative PCR and Western blot analysis revealed that THF regulates the activity of Jurkat cells and HT-29 cells via the NFκB and MAPK pathways under stimulated conditions. In the DSS-induced colitis model, oral administration of THF attenuated the manifestations of DSS-induced colitis, including a reduction in body weight, shrinkage of the colon, and enhanced expression of pro-inflammatory cytokines in the colon and mesenteric lymph nodes. These data suggest that THF alleviates DSS-induced colitis by modulating the activity of T cells and colon cells in vivo.

## 1. Introduction

There are two main subtypes of inflammatory bowel diseases (IBDs) of the gastrointestinal tract: Crohn’s disease (CD) and ulcerative colitis (UC). Although these diseases are chronic and are caused by an immune-mediated inflammatory response, there are fundamentally distinct differences between the two; CD mostly occurs in the ileum that represents non-uniform regions, whereas UC involves continuous inflammation of the colonic mucosa [1,2,3]. Effector T cells (Th1 and Th17) play an important role in intestinal inflammation by generating effector cytokines, such as interferonγ (IFNγ) and IL-17 [4]. Despite considerable improvements in IBD research involving the development of treatments for IBD over the last decade, therapeutic strategies using the simultaneous regulation of T cells and colon cell activity by small bioactive molecules remain insufficient.

During the first phase, primed naïve T cells in the mesenteric lymph node (MLN) are activated by dendritic cells and proliferate by generating IL-2. In the cytokine milieu, activated T cells are differentiated into effector T cells by the induction of master transcription factors, including T-bet for Th1 and RORγt for Th17 [5]. The differentiation of T cells into the Th1/Th17 axis is a critical event in chronic colitis, in which IFN-γ and IL-17 produced by effector T cells lead to chronic inflammation in the colon [6]. Colon cells are mainly stimulated with TNFα, which is generated by various immune-mediated cells in colitis conditions [7,8]. Activated colon cells produce TNFα, IL-1β, IL-6, and IL-8, which act as pro-inflammatory cytokines. Since T cells and colon cells are mainly responsible for colitis and intestinal inflammation, simultaneous modulation of these cell activities is highly efficient.

6,7,4′-trihydroxyflavanone (C_15_H_12_O_5_, THF) is a flavanone isolated from *Dalbergia odorifera* (Leguminosae) [9]. Leguminosae have traditionally been used as medicinal plants that grow in southern China. The bioactivities of this plant include anti-rheumatic, anti-epigastric pain, anti-swelling, and anti-ischemic effects [10]. We recently demonstrated that THF effectively blocks the progress of osteoblastogenesis, which protects against bone loss [9] and exerts an immunoprotective effect against methamphetamine-induced cytotoxicity on T cells [11]. Although THF has bioactive potential, it is unclear whether THF has a suppressive effect on T cells and colon cell activity. In addition, whether THF has a protective effect on colitis remains unknown.

In the present study, we investigated whether THF has a modulatory effect on the activity of T cells as well as colon cells. Jurkat cells and HT-29 cells were used for the in vitro study, respectively. To address the therapeutic potential of THF with underlying mechanisms, a DSS-induced colitis model was used. Reduced activity of T cells and colon cells by oral administration of THF was also elucidated in the MLN and colon tissues.

## 2. Results

### 2.1. THF Does Not Have a Negative Effect on the Viability of Jurkat Cells and HT-29 Cells 

**Figure 1:**
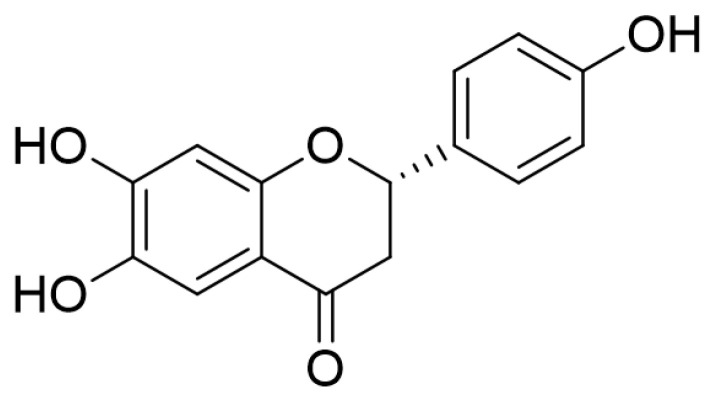
The chemical structure of 6,7,4′-trihydroxyflavanone (THF).

**Figure 2:**
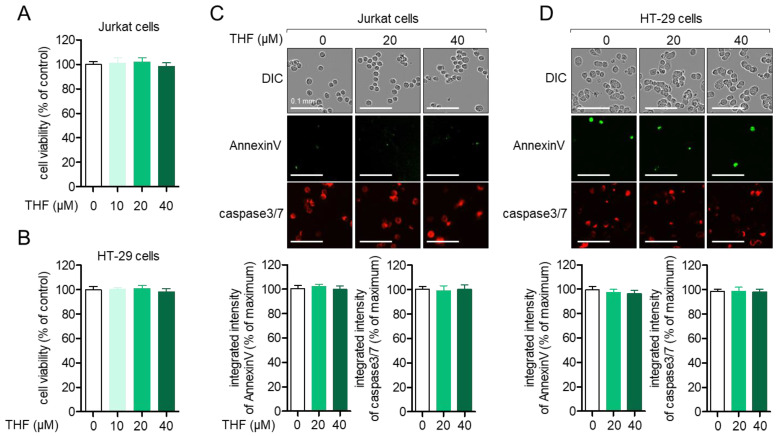
(**A**,**B**) Jurkat cells (**A**) or HT-29 cells (**B**) (5 × 10^3^/well, 96-well plates) were treated with the indicated concentration of THF (0–40 μM) for 24 h and an MTT assay was performed to determine cell viability. (**C**,**D**) Jurkat cells (**C**) or HT-29 cells (**D**) (5 × 10^3^/well, 96-well plates) were treated with 0, 20, and 40 μM of THF for 24 h and images of differential interference contrast (DIC), AnnexinV (green), and caspase 3/7 (red) were obtained by the IncuCyte imaging system. White bar indicates 0.1 mm. The integrated intensity of AnnexinV and caspase3/7 was calculated with the intensity of control cells and the percentage of maximum is presented in a bar graph. The mean value of three experiments ± SEM is presented.

Since the cytotoxic effect of small molecules can be one of the regulatory mechanisms on the activity of cells, we evaluated whether THF (Figure 1) induces cytotoxicity in Jurkat T cells and HT-29 colon cells. Results from the MTT assay showed that treatment with up to 40 μM of THF for 24 h did not lead to cytotoxicity in Jurkat (Figure 2A) and HT-29 cells (Figure 2B). To confirm whether treatment with THF is involved in the apoptotic pathway in these cells, the intensity of AnnexinV and caspase3/7 expressed from cells was assessed using the IncuCyte imaging system. Figure 2C shows that the integrated intensity of AnnexinV and caspase3/7 was not altered by treatment with up to 40 μM of THF. In HT-29 cells, treatment with THF did not change the intensity of annexin V and caspase3/7 (Figure 2D). These results suggest that incubation with up to 40 μM of THF does not have a negative effect on Jurkat T cells and HT-29 colon cells.

### 2.2. THF Regulates the Activity of Jurkat T Cells and HT-29 Cells in Stimulated Conditions

**Figure 3:**
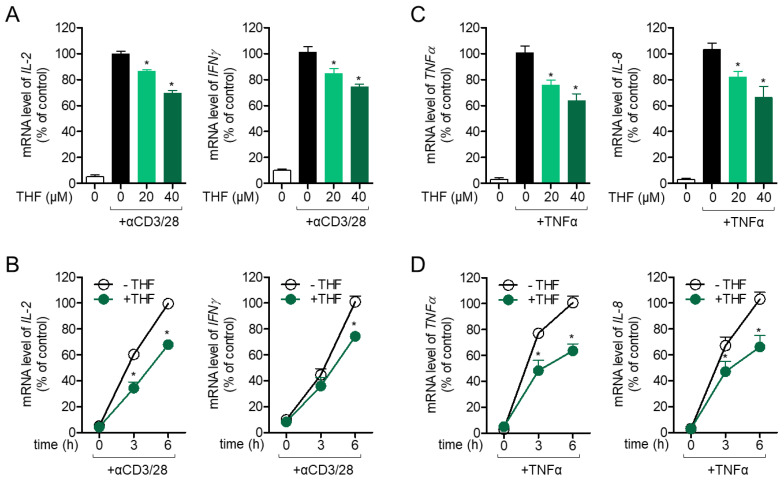
(**A**,**B**) Jurkat cells were pre-treated with the indicated concentration (0 to 40 μM) of THF (**A**) or 40 μM (**B**) for 1 h and stimulated with immobilized anti-CD3 antibodies (20 μg/mL) and soluble anti-CD28 antibodies (7 μg/mL) for 6 h (**A**) or the indicated time (**B**). The mRNA levels of IL-2 and IFNγ from the activated T cells were measured by quantitative real-time PCR. (**C**,**D**) HT-29 cells were pre-treated with the indicated concentration of THF (0 to 40 μM) (**C**) or 40 μM (**D**) for 1 h and stimulated with human TNFα recombinant protein (10 ng/mL) for 6 h (**C**) or the indicated time (**D**). The mRNA levels of TNFα and IL-8 from the activated HT-29 cells were measured by quantitative real-time PCR. The mRNA level of the indicated genes was normalized to the level of GAPDH. The mean value of three experiments ± SEM is presented. * *p* < 0.05 between the indicated group and mock-treated cells.

To determine whether treatment with THF has a modulatory effect on T cell activation, mRNA levels of *IL-2* and *IFNγ* were determined in activated T cells pre-treated with THF in a dose- or time-dependent manner. Figure 3A shows that pre-treatment with up to 40 μM THF considerably inhibited the mRNA levels of *IL-2* and *IFNγ*, which are activation markers of Jurkat T cells stimulated with anti-CD3/CD28 antibodies. *IL-2* and *IFNγ* downregulated by pre-treatment with 40 μM of THF were also assessed in a time-dependent manner (Figure 3B). The inhibitory effect of THF on the activity of HT-29 cells was evaluated, since HT-29 cell activation is mainly involved in intestinal inflammation. Figure 3C shows that pre-treatment with THF effectively modulated *TNF-α* and *IL-8* production, stimulated by TNF-α treatment in HT-29 cells. Results from time-dependent experiments confirmed that pre-incubation with THF suppressed the mRNA levels of *TNF-α* and *IL-8* in activated HT-29 colon cells (Figure 3D). These results suggest that THF regulates the activity of Jurkat and HT-29 cells under stimulated conditions.

### 2.3. Pre-Treatment with THF Suppresses MAPK Signaling Pathway via p65 Translocation in Activated Jurkat and HT-29 Cells

**Figure 4:**
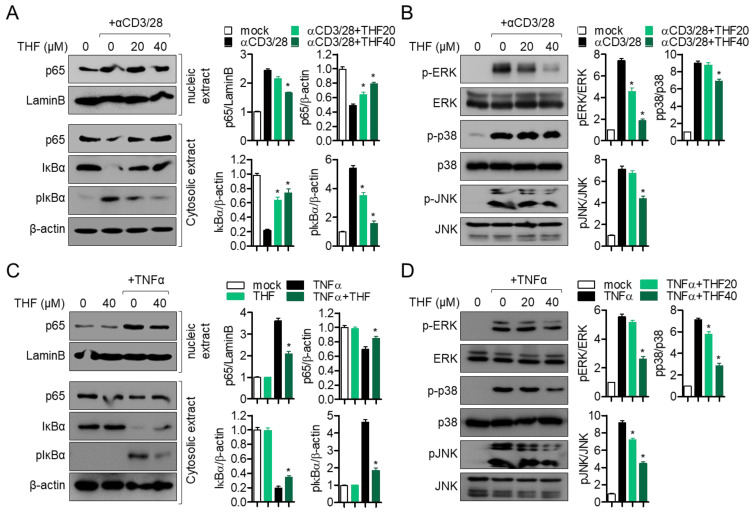
(**A**,**B**) Jurkat cells were pre-treated with the indicated concentration of THF for 1 h and stimulated with immobilized anti-CD3 antibodies (20 μg/mL) and soluble anti-CD28 antibodies (7 μg/mL) for 1 h (**A**) or 30 min (**B**). (**C**,**D**) HT-29 cells were pre-treated with 40 μM (**C**) or the indicated concentration (**D**) of THF for 1 h and stimulated with human TNFα recombinant protein (10 ng/mL) for 1 h (**C**) or 30 min (**D**). After lysis, the indicated proteins were detected by western blot analysis and normalized with LaminB for nuclear extract, β-actin for cytosolic extract, and total protein for each band of phosphorylated proteins. The mean value of three experiments ± SEM is presented. * *p* < 0.05 between mock-treated cells.

Since the NF-κB and MAPK pathways are the most important mechanisms for the activity of Jurkat cells and HT-29 cells, we explored whether THF affects the NF-κB and MAPK pathways in activated cells. Figure 4A shows that pre-treatment with THF mitigates p65 nuclear translocation in activated T cells by T cell receptor (TCR)-mediated stimulation. Degradation and phosphorylation of IκBα were also preserved by pre-treatment with THF. Inhibition of ERK, p38, and JNK phosphorylation was observed in activated T cells pre-treated with THF for 1 h (Figure 4B). In HT-29 cells, p65 nuclear translocation was significantly reduced by pre-treatment with THF (Figure 4C). Western blot results also revealed protected degradation and phosphorylation of IκBα by pre-treatment with THF. Figure 4D shows that the enhancement of phosphorylation levels of ERK, p38, and JNK by TNF-α was significantly downregulated by THF pre-treatment in HT-29 colon cells. These results suggest that the regulatory effects of THF on the activity of T cells and colon cells are associated with the suppression of p65 nuclear translocation and the MAPK pathway in vitro.

### 2.4. Oral Administration of THF Attenuates DSS-Induced Colitis in a Mouse Model

**Figure 5:**
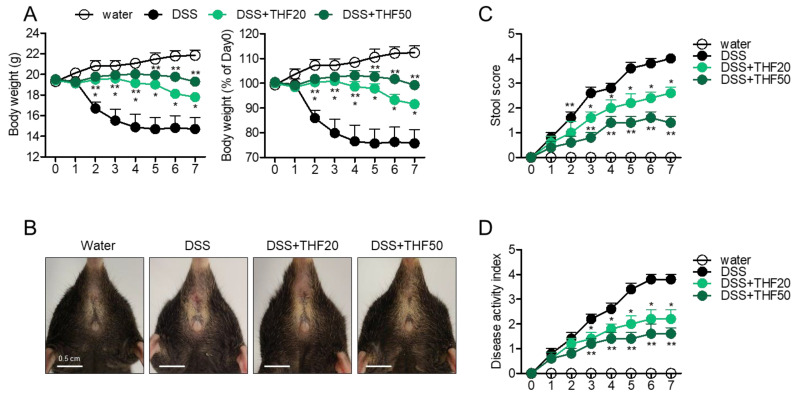
(**A**) Changes in the body weights of the experimental mice. (**B**) Representative anus pictures of each mouse group. (**C**) Changes in the stool scores of the experimental mice. White bar indicates 0.5 cm. (**D**) Changes in the disease activity index of the experimental mice. Water group: mice given drinking water, DSS group: mice given drinking water containing 2.5% DSS for seven days, DSS + THF20 group: mice given drinking water containing 2.5% DSS and orally administered 20 mg/kg THF every day for seven days, DSS + THF50 group: mice given drinking water containing 2.5% DSS and orally administered 50 mg/kg THF every day for seven days. The mean value of five mice ± SEM is presented. * *p* < 0.05 between the DSS and DSS + THF20 group; ** *p* < 0.05 between the DSS and DSS + THF50 group.

We investigated whether THF has a protective effect in a DSS-induced colitis mouse model based on the mechanism we elucidated in vitro. To induce the systemic effect of THF on intestinal inflammation, oral administration of two doses (20 and 50 mg/kg) was undertaken. During the induction of colitis via oral consumption of drinking water containing 2.5% DSS for seven days, the pathogenic progress was monitored by measuring body and stool shape. Figure 5A shows that the body weight of colitis mice gradually decreased for seven days, but oral administration of THF partially blocked the reduction in body weight in a dose-dependent manner. Figure 5B shows that oral administration of THF mitigated colitis manifestations, including rubefaction around the anus skin and bleeding. During the experiment, the shape of the stool revealed significant improvements after oral administration of THF in a dose-dependent manner (Figure 5C). Colitis mice treated with THF also exhibited an ameliorated disease activity index in a dose-dependent manner (Figure 5D). These results suggest that the oral administration of THF attenuates the symptoms of DSS-induced colitis in vivo.

### 2.5. Oral Administration of THF Suppresses DSS-Induced Intestinal Inflammation

**Figure 6:**
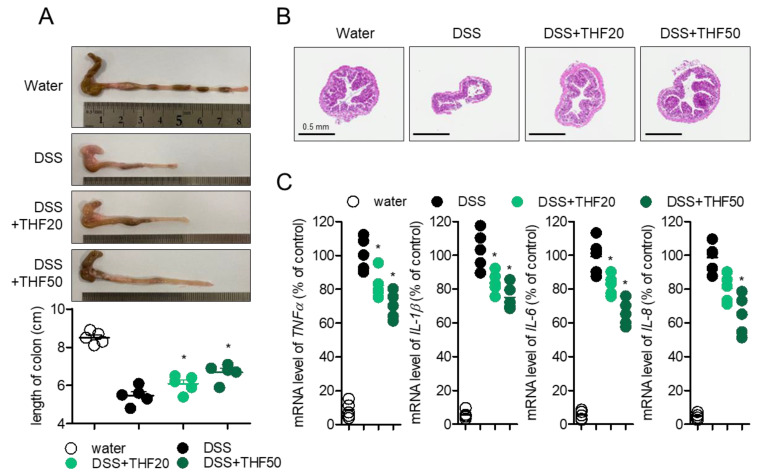
(**A**) Representative images of colons from each mouse group and the lengths of the colons. (**B**) Representative H&E images of colons from each mouse group, axially sectioned. Black bars indicates 0.5 mm. (**C**) mRNA levels of the indicated genes in the colon tissues from each mouse group. mRNA levels of the indicated genes were normalized with those of GAPDH. Water group: mice given drinking water, DSS group: mice given drinking water containing 2.5% DSS for seven days, DSS + THF20 group: mice given drinking water containing 2.5% DSS and orally administered 20 mg/kg THF every day for seven days, DSS + THF50 group: mice given drinking water containing 2.5% DSS and orally administered 50 mg/kg THF every day for seven days. The mean value of five mice ± SEM is presented. * *p* < 0.05 between the DSS mouse group.

To investigate the protective effect of THF on DSS-induced colitis in more detail, colons were removed to compare their lengths, since colon length shrinkage is a typical manifestation of colitis. Figure 6A shows that colons isolated from colitis mice were diminished compared to colons from control mice, but oral administration of THF partially recovered the lengths of colons in a dose-dependent manner. The histological alteration in the intestinal structure was determined by monitoring the axial sections of colons stained with hematoxylin and eosin (H&E). Shrunken colons with crypt architecture and inflammatory cell infiltration were observed in DSS-induced colitis mice, but improved colons were observed in colitis mice with the oral administration of THF (Figure 6B). For molecular evaluation, mRNA levels of pro-inflammatory cytokines from intestinal epithelial cells were detected by quantitative PCR to determine whether the oral administration of THF inhibited inflammation in the colon. Figure 6C shows enhanced production of pro-inflammatory cytokines, including *TNFα*, *IL-1β*, *IL-6*, and *IL-8*; THF treatment partially suppressed these manifestations in colon tissues. These results demonstrate that THF administration regulates intestinal inflammation in a DSS-induced colitis model.

### 2.6. Oral Administration of THF Blocks Th1/Th17 Cells to Produce the Effector Cytokines in a DSS-Induced Colitis Model

**Figure 7:**
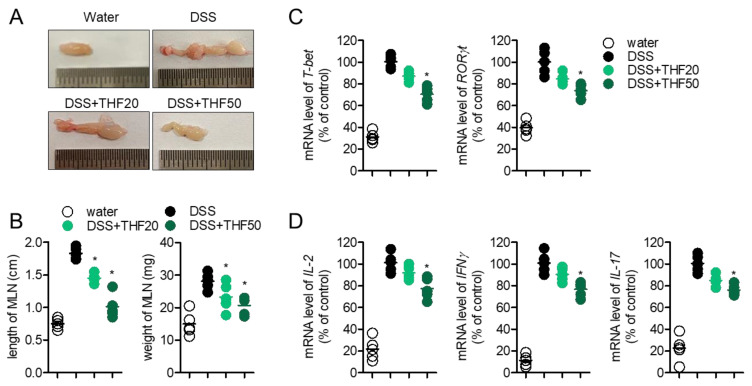
(**A**) Representative images of the mesenteric lymph node (MLN) from each mouse group. (**B**) The length and weight of the MLN from each mouse group. (**C**,**D**) mRNA levels of the indicated genes of the MLN from each mouse group. mRNA levels of the indicated genes were normalized with those of GAPDH. Water group: mice given drinking water, DSS group: mice given drinking water containing 2.5% DSS for seven days, DSS + THF20 group: mice given drinking water containing 2.5% DSS and orally administered 20 mg/kg THF every day for seven days, DSS + THF50 group: mice given drinking water containing 2.5% DSS and orally administered 50 mg/kg THF every day for seven days. The mean value of five mice ± SEM is presented. * *p* < 0.05 between the DSS mouse group.

The MLN is an important organ for modulating the immunological response in the colon. Since T cells play a pivotal role in colitis, we explored whether the oral administration of THF affects T cell activity, including differentiation into Th1/Th17 and the production of effector cytokines in DSS-induced colitis. Figure 7A shows that the size of MLN isolated from colitis mice was augmented compared to that in control mice, but oral administration of THF partially decreased the size of MLN in colitis. The length and weight of MLN were effectively reduced by the oral administration of THF in colitis (Figure 7B). To understand whether THF administration systemically affects T cell differentiation into Th1 and Th17, mRNA levels of master transcription factors of Th1 and Th17, including *T-bet* and *RORγt*, were assessed in the MLN by quantitative PCR analysis. Figure 7C shows that the expression of *T-bet* and *RORγt* was slightly downregulated in the MLN tissue of colitis mice with the oral administration of THF, compared to that in colitis mice in a dose-dependent manner. The mRNA levels of effector cytokines, including IL-2, IFNγ, and *IL-17*, were also detected, to confirm whether THF administration influences T cell activity in a colitis model. Figure 7D shows that the oral administration of THF partially suppresses the production of effector cytokines from activated T cells in colitis. These results suggest that the oral administration of THF slightly blocks T cell differentiation into Th1/Th17 and the production of effector cytokines in a colitis model.

## 3. Discussion

In the current study, we evaluated the suppressive effect of THF on the activity of T cells and intestinal epithelial cells in vitro with our cytotoxicity [9], and suggested its therapeutic potential for colitis by downregulating intestinal inflammation and T cell differentiation into effector cells. We found that the regulation of T cells and colon cell activity by THF is due to modulation of the NFκB pathway by inhibiting p65 nuclear translocation, as shown in RAW264.7 cells [9], degradation, and phosphorylation of IκBα. Based on the underlying mechanism at the cellular level, we found that oral administration of THF attenuates colitis manifestations, including the reduction in body weight, bloody stools, shrinkage of colons, and expression of pro-inflammatory cytokines in colon tissues. Our results revealed that this improvement by THF is correlated with the downregulated activity of T cells and colon cells in vivo.

T cell differentiation into effector cells, including Th1/Th17, is a key event in colitis [12,13,14]. To differentiate these into effector cells, naïve T cells are primed by antigen-presenting cells capturing specific antigens. In the course of TCR-major histocompatibility complex ligands, TCR-mediated signaling induces T cell activation and IL-2 is generated, which plays a critical role in the proliferation and differentiation of T cells. In the present study, we showed that pre-treatment with THF efficiently inhibited T cells activated by anti-CD3/CD28 antibodies (Figure 3A,B). In addition, we revealed that the mRNA levels of *IL-2* and *IFNγ* were mitigated by the oral administration of THF in a colitis model (Figure 7D). The requirement of IL-2 for Th1 differentiation [15] suggests that the regulation of T cell activation is a potent clinical strategy for colitis.

IL-6 is a pro-inflammatory cytokine produced by macrophages, T cells, B cells, and intestinal epithelial cells [16]. Several studies have elucidated the effects of IL-6 on T cell differentiation, which promotes polarization into Th17 through JAK-STAT3 signaling [17,18]. Our results from animal experiments show that the oral administration of THF significantly reduced the mRNA levels of *IL-6* in colon tissue (Figure 6C) and mRNA levels of *RORγt* and *IL-17* in the MLN (Figure 7C,D). These results suggest that the reduction in IL-6 expression in colon cells is linked to suppressed differentiation into Th17 and its activity to produce IL-17. Further studies should investigate how THF controls IL-6 expression at the molecular level and its physiological significance in chronic diseases.

The MLN is an important organ that acts as a regulator of mucosal inflammation by controlling homeostasis between tolerance and immunity [5]. It is considered an inductive site for intestinal dendritic cells to prime naïve T cells and help them differentiate into effector cells, including Th1/Th17. For immunological events during colitis in terms of T cell activation and differentiation, the MLN is the most important organ for simultaneous immune responses. In the present study, we found that the length and weight of the MLN significantly increased in colitis but was suppressed by the oral administration of THF (Figure 7A,B). This suggests that THF systemically affects intestinal inflammation and plays an inhibitory role in other immune cells. The underlying modulatory mechanism of THF on other immune cells, including B cells, macrophages, and neutrophils, should be investigated further.

The flavanone family, which is a member of THF, has been investigated regarding its ability to control the colitis or colonic inflammation with various mechanisms. Naringin has been shown to suppress acetic acid-induced colitis via regulation of endogenous balance between oxido and nitrosative in rats [19]. It has been investigated that diplacone and mimulone protect rats from DSS-induced colitis through increments in antioxidant activity and modulation of metalloproteinase activity [20]. A recent report has evaluated the protective effect of pinocembrin on colitis, whereby it suppresses gut microbiota and toll like receptor 4/myeloid differentiation factor 2/NF-κB pathway but enhances the intestinal barrier by upregulating the expression of tight junction proteins [21]. Since the literature reports on the potent bioactivities of flavanone family on the attenuation of colitis, further studies should look at whether THF has an antioxidant activity, modulates metalloproteinase activity or increases intestinal barrier function in colonic inflammation conditions.

To examine the protective effect of THF on T cell-mediated colitis and its underlying mechanism, we used two cell lines: Jurkat T cells and HT-29 colon cells. These two immortal cell lines are widely used for the mechanistic investigation of T lymphocytes and intestinal epithelial cells, since they possess the most of biological characteristic of T cells and colon cells [22,23,24]. Despite their convenience and easiness, it is also obvious that these two cells are categorized as cancer cells: Jurkat T cells are considered as leukemia and HT-29 cells are studied as colorectal adenocarcinoma in the cancer research field [25,26]. To overcome the limitations of the current study, we exhibited several results from colon tissues and MLNs to show the protective effect of THF on colitis that involve mRNA level of pro-inflammatory cytokines, length of tissues and histological analysis by staining with H&E (Figure 6 and Figure 7). Results from animal experiments support our hypothesis that THF has an attenuating effect on colitis. Further studies should include a culture system which closely reflects the characteristics of the in vivo intestinal epithelium, such as 3D co-culture model of Caco-2 and HT29-MTX cells model or isolation of primary T lymphocytes from healthy mice.

## 4. Materials and Methods

### 4.1. Cells

Jurkat human T cells (KCLB No. 40152) and HT-29 human colon cells (KCLB No. 30038) were purchased from the Korean Cell Line Bank (Seoul, Korea). The cells were identified by KCLB before distribution. Jurkat cells were cultured in RPMI medium (Welgene, Gyeongsan-si, Korea) and HT-29 cells were cultured in DMEM (Welgene, Gyeongsan-si, Korea) supplemented with 10% fetal bovine serum, penicillin G (1 X), L-glutamine (2 mM), and streptomycin (1×) at 37 °C in a humidified incubator containing 5% CO_2_. Both cell lines were maintained within passage #12 to obtain accurate and consistent experimental results.

### 4.2. Mice

Eight-week-old female C57BL/6J mice were purchased from Samtako Bio (Osan, Korea) and housed under specific pathogen-free (SPF) conditions. All experiments were approved by the Animal Care and Use Committee of the College of Pharmacy, Keimyung University (approval number: KM2020-004, 2 June 2020).

### 4.3. Isolation of THF from D. odorifera

THF (M.W: 270.24) was isolated from *D. odorifera* as previously reported [9]. Briefly, *D. odorifera* was provided by the Herbal Medicine Cooperative Association (Jeonbuk Province, Republic or Korea). Dried *D. odorifera* (20 kg) was extracted four times with 100% EtOH. The filtered EtOH extract (2.416 kg) was concentrated and partitioned with CH_2_Cl_2_. The CH_2_Cl_2_-soluble fraction (200 g) was collected and subjected to chromatography on a silica gel column with n-hexane-EtOAc (1:0 to 0:1) to obtain five fractions (Fr. 1–5). Among the five fractions, Fr. 3 (120 g) was isolated on a Sephadex LH-20 column with a mixture of solvents (MeOH: H_2_O = 9:1) and four fractions were collected (Fr. 3_1–3_4). Among the four fractions, Fr. 3_3 (30 g) was isolated on a Sephadex LH-20 column with a mixture of solvents (EtOAc:MeOH = 4:1) and loaded on a silica gel column with a gradient mixture of solvents from 100% n-hexane to 100% EtOAc to collect compound 6 (120 mg). Compound 6 was identified as THF using ^1^H and ^13^C nuclear magnetic resonance (NMR) spectral data based on a previous study [27] and its purity was determined to be 98.8% by NMR (JEOL JNM-ECA 500).

### 4.4. Reagents and Antibodies

Antibodies against human CD3 and CD28 for the stimulation of T cells were purchased from BioXcell (West Lebanon, NH, USA). Human TNFα recombinant protein was obtained from PeproTech EC Ltd. (London, UK). TRIZOL reagent for RNA isolation, MTT powder (1-(4,5-dimethylthiazol-2-yl)-3,5-diphenylformazan), and radioimmunoprecipitation assay (RIPA) buffer for cell lysis were provided by Sigma Chemical Co. (St. Louis, MO, USA). Staining reagents for AnnexinV (green) and caspase3/7 (red) were purchased from Essen Bio (Ann Arbor, MI, USA). SYBR Premix Ex Taq was obtained from TaKaRa (Shiga, Japan). Anti-p65, anti-LaminB, anti-IκBα, anti-phosphorylated IκBα, anti-phosphorylated ERK, anti-phosphorylated p38, anti-p38, anti-phosphorylated JNK, and anti-JNK were provided by Cell Signaling Technology (Danvers, MA, USA). Antibodies against actin and ERK were purchased from Santa Cruz Biotechnology (Dallas, TX, USA). ECL Western blotting detection reagents were obtained from Thermo Fisher Scientific (Waltham, MA, USA). The RT PreMix kit was provided by Enzynomics (Daejeon, Korea). DSS was purchased from MP Biomedicals (Irvine, CA, USA).

### 4.5. MTT Assay

Seeded Jurkat cells (5 × 10^3^/well, 96-well plate) or HT-29 cells (5 × 10^3^/well, 96-well plate) were treated with the indicated concentrations of THF for 24 h. The medium was discarded and cells were incubated with culture medium containing 500 μg/mL of MTT for 2 h at 37 °C. After the removal of the supernatants, 160 μL of DMSO was added to dissolve the formazan crystals and mixed gently. To obtain the absorbance at 540 nm, the plate was read, and cell viability was calculated by comparing the absorbance of obtained control cells (% of control).

### 4.6. Measurement of AnnexinV and Caspase3/7 by the IncuCyte Imaging System

Seeded Jurkat cells (5 × 10^3^/well, 96-well plate) or HT-29 cells (5 × 10^3^/well, 96-well plate) were stained with staining reagents (AnnexinV and caspase3/7) and treated with the indicated concentration of THF (0, 20, or 40 μM) for 24 h. Images of cells were obtained using the IncuCyte^®^ imaging system, and the intensity of Annexin V and caspase3/7 was also obtained. Integrated intensity of AnnexinV and caspase3/7 was calculated by comparing the intensity of control cells (% of control).

### 4.7. Real-Time Quantitative PCR

To analyze the mRNA levels of the indicated genes, total RNA was isolated from Jurkat cells, HT-29 cells, colon tissues, or MLN tissues treated under the indicated conditions. Reverse transcription of total RNA was performed to obtain cDNA. Primers used for each gene were as follows (forward and reverse primers, respectively): human *IL-2*, 5′-CAC GTC TTG CAC TTG TCA C-3′ and 5′-CCT TCT TGG GCA TGT AAA ACT-3′; human *IFNγ*, 5′-TGG CTT TTC AGC TCT GCA TC-3′ and 5′-CCG CTA CAT CTG AAT GAC CTG-3′, human *TNFα*, 5′-CCT ACC AGA CCA AGG TCA AC-3′ and 5′-AGG GGG TAA TAA AGG GAT TG-3′, human *IL-8*, 5′-GTG CAG TTT TGC CAA GGA GT-3′ and 5′-TTA TGA ATT CTC AGC CCT CTT CAA AAA-3′, human *GAPDH*, 5′-CGG AGT CAA CGG ATT TGG TCG TAT-3′ and 5′-AGC CTT CTC CAT GGT GGT GAA GAC-3′, mouse *TNFα*, 5′-GGC AGG TCT ACT TTG GAG TCA TTG C-3′ and 5′-ACA TTC GAG GCT CCA GTG AAT TCG G-3′, mouse *IL-1β*, 5′-ATA ACC TGC TGG TGT GTG AC-3′ and 5′-AGG TGC TGA TGT ACC AGT TG-3′, mouse *IL-6*, 5′-CCG GAG AGG AGA CTT CAC AG-3′ and 5′-GGA AAT TGG GGT AGG AAG GA-3′, mouse *IL-8*, 5′-ATG GCT GCT CAA GGC TGG TC-3′ and 5′-AGG CTT TTC ATG CTC AAC ACT AT-3′, mouse *T-bet*, 5′-AGA AGG ACG GCG AAT GTT-3′ and 5′-GGG TGG ACA TAT AAG CGG TTC-3′, mouse *RORγt*, 5′-TGT CCT GGG CTA CCC TAC TG-3′ and 5′-GTG CAG GAG TAG GCC ACA TT-3′, mouse *IL-2*, 5′-TGA GCA GGA TGG AGA ATT ACA GG-3′ and 5′-GTC CAA GTT CAT CTT CTA GGC AC-3′, mouse *IFNγ*, 5′-TCA AGT GGC ATA GAT GTG GAA GAA-3′ and 5′-TGG CTC TGC AGG ATT TTC ATG-3′, mouse *IL-17*, 5′-TCC CCT CTG TCA TCT GGG AAG-3′ and 5′-CTC GAC CCT GAA AGT GAA GG-3′, and mouse *GAPDH*, 5′-GCA CAG TCA AGG CCG AGA AT-3′ and 5′-GCC TTC TCC ATG GTG GTG AA-3′. For quantitative PCR, amplification was performed in a DNA Engine Opticon 1 continuous fluorescence detection system using SYBR Premix Taq. The total reaction volume was 10 μL containing 0.1 μg of cDNA, and each PCR reaction was performed using the following conditions: 95 °C for 30 s, 60 °C for 30 s, and plate read for 40 cycles followed by 7 min of extension at 75 °C. Melting curve analysis was performed to characterize the dsDNA product by slowly increasing the temperature (0.2 °C/s) from 60 to 98 °C with fluorescence data collected at 0.2 °C intervals. mRNA levels of genes were normalized to mRNA levels of *GAPDH* and presented as % of the maximum. The maximum percentage was calculated using the following equation

% of maximum = 2^−ΔΔCT^ × 100

where ΔΔCT = (CT_target_ − CT_GAPDH_) at maximum − (CT_target_ − CT_GAPDH_).

### 4.8. Western Blot Analysis

Jurkat and HT-29 cells treated under the indicated conditions were harvested for lysis in RIPA buffer containing 1 × phosphatase inhibitor on ice for 18 min. Lysates were then centrifuged at 15,000× *g* at 4 °C for 17 min, and 40 μg of the lysate was loaded on 8–12% SDS–PAGE gels for separation. Proteins were transferred onto PVDF membranes and the membranes were blocked with 0.1% TBS-T containing 5% skim milk for 2 h. After blocking, membranes were incubated with the indicated primary antibodies in 0.1% TBS-T containing 3% skim milk overnight (1:1000 ratio) at 4 °C. The following day, excess antibodies were removed by washing the membrane three times with 0.1% TBS-T and were incubated with 0.1 μg/mL peroxidase-labeled secondary antibodies (against rabbit or mouse) for 2 h at RT. After three washes with 0.1% TBS-T, bands were detected with ECL Western blot detection reagents with an ImageQuant LAS 4000 (GE Healthcare, Chicago, IL, USA).

### 4.9. DSS-Induced Colitis Model 

The colitis model was induced by the administration of drinking water containing DSS. Twenty mice were grouped into four groups, as follows: mice given drinking water, mice given drinking water containing 2.5% DSS for seven days, mice given drinking water containing 2.5% DSS and orally administered 20 mg/kg THF every day for seven days, and mice given drinking water containing 2.5% DSS and orally administered 50 mg/kg THF every day for seven days (*n* = 5 mice/group). Daily changes in body weight and stool shape were monitored for seven days. The disease activity index was used to determine the clinical progression of colitis according to the reported criteria [28]. The disease activity index involves the score of relative body weight loss, shape of stool, and bleeding around the anus and stool. The scoring criteria were as follows: weight loss: 0 (no loss), 1 (1–5%), 2 (5–10%), 3 (10–20%), and 4 (more than 20%); stool form: 0 (normal), 2 (loose stool), and 4 (diarrhea); and bleeding: 0 (no blood), 1 (Hemoccult positive), 2 (Hemoccult positive and visual pellet bleeding), and 4 (bleeding around the anus).

### 4.10. H&E Staining 

Colons were collected and subjected to histological analysis after the mice were euthanized. The removed colons (0.5 cm) were fixed in 10% paraformaldehyde and embedded in paraffin. Embedded tissues were sliced (5-μm-thick axial sections) and deparaffinized. Deparaffinized tissues were stained with H&E for histological analysis.

### 4.11. Statistical Analysis

Mean values ± SEM were calculated from the data collected from three independent experiments performed on separate days and are presented in bar graphs. One-way ANOVA was used to determine significance (*p* value). * indicates differences between the two indicated groups considered significant at *p* < 0.05.

## 5. Conclusions

In conclusion, oral administration of THF attenuated the manifestations of DSS-induced colitis, including reduction in body weight, shrinkage of the colon, and enhanced expression of pro-inflammatory cytokines in the colon and mesenteric lymph nodes. These data suggest that THF alleviates DSS-induced colitis by modulating the activity of T cells and colon cells in vivo.

## Data Availability

The data presented in this study are available on request from the corresponding author.

## Abbreviations

THF	6,7,4′-tryhydroxyflavanone
TCR	T cell receptor
NF-κB	Nuclear factor kappa B
IBD	Immune bowel disease
